# ST3GAL3 Promotes the Inflammatory Response of Fibroblast-Like Synoviocytes in Rheumatoid Arthritis by Activating the TLR9/MyD88 Pathway

**DOI:** 10.1155/2022/4258742

**Published:** 2022-11-10

**Authors:** Liming Xu, Xuegang Niu, Yifan Liu, Lining Liu

**Affiliations:** ^1^Department of Rheumatism and Immunology, Liaocheng People's Hospital, Liaocheng, 252000 Shandong, China; ^2^Department of Orthopedics, Liaocheng Hospital of Traditional Chinese Medicine, Liaocheng, 252000 Shandong, China

## Abstract

This study is aimed at investigating the role of *β*-galactoside-*α*2,3-sialyltransferase III (ST3GAL3) in fibroblast-like synoviocytes (FLS) in rheumatoid arthritis (RA), as well as its potential mechanism of action. The Gene Expression Omnibus (GEO) database and gene set enrichment analysis (GSEA) were used to analyse the expression of *ST3GAL3* and the enrichment signalling pathways associated with *ST3GAL3* in RA. The effects of ST3GAL3 on tumour necrosis factor- (TNF-) *α* and interleukin- (IL-) 1*β*-treated MH7A cells were determined using methyl thiazolyl tetrazolium (MTT), transwell, and enzyme-linked immunosorbent assays (ELISA). The expression of proliferation-associated proteins and Toll-like receptor (TLR) pathway-enriched proteins was analysed using western blotting. As a main result, *ST3GAL3* was screened as an overlapping upregulated gene from GSE101193 and GSE94519 datasets. ST3GAL3 expression in MH7A cells significantly increased with increasing treatment time with TNF-*α* or IL-1*β*. TLR9/myeloid differentiation primary response protein 88 (MyD88) is a downstream activation pathway of ST3GAL3. ST3GAL3 overexpression promoted MH7A cell proliferation and migration. Additionally, ST3GAL3 overexpression upregulated the expression of proliferation-associated proteins (cyclinD, cyclinE, and proliferating cell nuclear antigen) and TLR pathway enrichment factors (TLR9 and MyD88) and increased the production of matrix metallopeptidase (MMP) 1, MMP3, interleukin- (IL-) 6, and IL-8, whereas si-ST3GAL3 had the opposite effect. The addition of TLR9 agonists (CpG 2216 and CpG 2006) reversed the effects of si-ST3GAL3 on MH7A cell proliferation, migration, and inflammation. TLR9-specific siRNA reversed the effects of ST3GAL3 overexpression on MH7A cell proliferation, migration, and inflammation. In conclusion, ST3GAL3 is likely involved in RA pathogenesis by activating the TLR9/MyD88 pathway.

## 1. Introduction

Rheumatoid arthritis (RA) is a complicated, chronic, and systemic autoimmune disease. According to statistics, the incidence of RA is increasing annually worldwide, with a morbidity of 0.5–2%, especially in developing countries [1]. Women are at a significantly higher risk of RA [2]. RA is characterised by synovial hyperplasia and injury, swelling and pain of the joints, aggressive joint inflammation, and progressive destruction of the cartilage synovium [3]. It not only affects the joints of patients but also impacts the majority of apparatus and systems such as the skeletal, lung, nervous, and cardiovascular systems [4–6]. RA is considered a major cause of physical disability that seriously affects the quality of life of patients, increases medical costs, and imposes a serious economic burden on society [7]. Therefore, there is an urgent need to identify effective treatments to prevent the occurrence of RA.

To date, the detailed molecular mechanism of RA morbidity is still largely unclear resulting in uncertainty and difficulties in finding a reliable treatment. Fibroblast-like synoviocytes (FLS) are mesenchymal stem cells that act like fibroblasts [8]. FLS can naturally secrete a large number of matrix metalloproteinases (MMPs) and proinflammatory cytokines that have significant effects on the progressive destruction of articular cartilage [9, 10]. FLS can be induced by tumour necrosis factor- (TNF-) *α* or interleukin- (IL-) 1*β* and exhibit biological behaviours similar to tumour cells, such as resistance to apoptosis and aggressive proliferation [11, 12]. Therefore, the inhibition of FLS proliferation may have a therapeutic effect on RA, providing a reasonable and effective method for the treatment of RA.

FLS play a vital role in the pathological processes of RA by mediating several relevant pathways [8]. The Toll-like receptor (TLR) signalling pathway involves five adaptor proteins, including myeloid differentiation primary response protein 88 (MyD88) [13]. TLRs are a type of pattern recognition receptor, and thus far, 10 hypotypes have been confirmed in humans (TLR1-10) [14]. TLRs play a key role in regulating of innate immunity. Recently, many studies have revealed the importance of TLRs in autoimmune diseases, including RA [15]. Furthermore, TLR9 is markedly highly expressed in RA patients with active disease [16]. The addition of CpG-ODNs (TLR9 agonists) could alleviate joint inflammation by enhancing the production of TNF-*α* or IL-1*β* and restraining synovial neutrophil infiltration [17]. To date, the TLR9/MyD88 pathway has not been studied in detail in the context of RA progression.

*β*-Galactoside-*α*2,3-sialyltransferase III (ST3GAL3) is a pivotal enzyme in the biosynthesis of sialyl Lewis X (sLeX) oligosaccharides. Studies have shown that the expression of ST3GAL3 is related to the differentiation, progression, and metastasis of extrahepatic cholangiocarcinoma and the secondary recurrence of gastric carcinoma [18]. IL-1*β* promotes ST3GAL3 expression, leading to the glycosylation pattern of pancreatic ductal adenocarcinoma cells, which then promotes the malignancy of pancreatic tumours [19]. Furthermore, ST3GAL3 plays an important role in sustaining advanced cognitive function, regulating brain development, and participating in the inflammatory process [20, 21]. However, the molecular mechanism of ST3GAL3 in RA progression has not yet been elucidated.

In this study, *ST3GAL3* was screened from the Gene Expression Omnibus (GEO) database as a gene upregulated during RA. Considering its role in regulating the inflammatory process, we aimed to study its effect on synovial inflammation in RA and attempted to reveal its regulation with the TLR9/MyD88 signalling pathway. The findings of this study provide new insights into the pathogenesis of RA.

## 2. Materials and Methods

### 2.1. Bioinformatics Analysis

The expression profile datasets of RA patients were obtained from the publicly available GEO database (http://www.ncbi.nlm.nih.gov/geo). The expression of *ST3GAL3* was analysed using the GEO databases (GSE94519 and GSE101193). Microarray screening of GSE94519 was performed in the serum of three female patients with RA and three healthy female controls. Microarray screening of GSE101193 was performed in the peripheral blood mononuclear cells (PBMCs) of 27 female RA patients and 27 corresponding healthy controls. Patients with RA and healthy controls did not significantly differ with respect to the mean age or sex distribution. Gene set enrichment analysis (GSEA) was performed to confirm the enriched signalling pathways associated with *ST3GAL3* in RA. The Kyoto Encyclopedia of Genes and Genomes (KEGG) pathway enrichment analysis was performed using the clusterProfiler package in R.

### 2.2. MH7A Cell Culture

The human RA-derived FLS cell line (MH7A) was obtained from the RIKEN Cell Bank (Tsukuba, Japan). The MH7A cells were cultivated in Roswell Park Memorial Institute- (RPMI-) 1640 medium (Hyclone; GE Healthcare, UT, USA) supplemented with 1% streptomycin, 1% penicillin (Beijing Solarbio Science & Technology Co., Ltd., Beijing, China), and 10% foetal bovine serum (FBS, Thermo Fisher Scientific, Inc., MA, USA) under relatively damp conditions of 95% air 5% CO_2_, at 37°C. The medium was changed every 2 days, and the cells were passaged once the confluence reached 80%.

### 2.3. Drug Treatment

When MH7A cells were cultured to passage 3–4, they were used for drug treatment according to a previous study [22]. In brief, the MH7A cells were cultivated in sterile 96-well plates (5 × 10^3^ per well) in RPMI-1640 medium supplemented with 1% FBS and 1 mM pyruvate and then treated with TNF-*α* at 5 ng/mL (R&D Systems) or IL-1*β* at 20 ng/mL (R&D Systems, Inc.) to simulate local inflammation for 0–48 h at 37°C. Untreated MH7A cells were used as controls. 3′-sLeX (*α*-NeuNAc-(2→3)-*β*-D-Gal-(1→4)(*α*-L-Fuc-[1→3])-D-GlcNAc) was purchased from Sigma-Aldrich (St. Louis, MO, USA). MH7A cells were treated with 0.5 M 3′-sLeX for 24 h.

### 2.4. Cell Transfection

*ST3GAL3* siRNA (5′-GACAAACACTAGGCTCAGAGT-3′), siRNA scrambled negative control (5′-GCAATGAGAGCACCGATCAAT-3′), overexpression vector of *ST3GAL3*, and empty vectors were purchased from Ribobio (Guangzhou, China). Specific siRNA for *TLR9* (5′-GCAACTGGCTGTTCCTGAAGT-3′) and its scrambled negative control (5′-GCATGGTACGCGTCTGATCTA-3′) were also purchased from Ribobio. The MH7A cell line (2 × 10^4^ cells/well) was cultivated in 6-well plates in RPMI-1640 medium supplemented with 10% FBS for 24 h. Then, when MH7A cells grew to 80% confluence, the siRNAs and overexpression plasmids were transfected into MH7A cells using Lipofectamine 2000 reagent (Thermo Fisher Scientific), according to the manufacturer's instructions.

CpG oligonucleotides (CpG-ODNs) are considered effective agonists of TLR9. To confirm the effect of TLR9 on ST3GAL3 expression, MH7A cells transfected with si-ST3GAL3 in medium were added to two different CpG-ODNs, CpG 2216, or CpG 2006 (5 *μ*g/mL), as described in a previous study [23]. After 48 h of transfection, the MH7A cells were collected for subsequent experiments.

### 2.5. Real-Time Quantitative RT-PCR (qRT-PCR)

TRIzol reagent (Invitrogen, CA, USA) was used to extract total RNA from MH7A cells, according to the manufacturer's instructions. Then, 500 ng of total RNA was reverse-transcribed using PrimeScript RT reagent kit (perfect real-time kit, Takara, Japan). After cDNA amplification using a real-time PCR kit (Takara, Japan), qRT-PCR was performed to detect the expression of ST3GAL3 using the SYBR PremixEx Taq II kit (Takara, Japan) on a 7500 Real-Time PCR System (Applied Biosystems, USA). The reaction conditions were as follows: predenaturation, 95°C, 3 min; denaturation, 96°C, 15 s; and annealing, 58°C, 30 s, 40 cycles. The final relative expression of ST3GAL3 was calculated using the 2^*ΔΔ*Ct^ method and was normalised to that of *GAPDH*. The primers are as follows: *GAPDH*-F, 5′-GGAGCCAAAAGGGTCATC-3′; *GAPDH*-R, 5′-CCAGTGAGTTTCCCGTTC-3′; *ST3GAL3*-F, 5′-AAAACGACACTGCGCATCAC-3′; and *ST3GAL3*-R, 5′-TCGAGTGGCCACAGATTTCC-3′.

### 2.6. MTT Assay

After transfection, cell proliferation was evaluated using the methyl thiazolyl tetrazolium (MTT) assay. Briefly, transfected MH7A cells were cultivated in 96-well plates (4 × 10^3^ cell per well). Subsequently, 20  *μ*L of MTT solution (Beyotime, Shanghai, China) was added to each well. After hatching for 4 h at 37°C, 100 *μ*L of dimethyl sulfoxide (DMSO) was added to each well, and the 96-well plates were incubated and placed on a microoscillator until the crystals disappeared. The absorbance of each well was measured at 570 nm using a microplate reader (Multiskan Go, Thermo Fisher Scientific).

### 2.7. Transwell Assay

The migration ability of transfected MH7A cells was assessed using transwell chambers with 8 *μ*m porous membranes coated with Matrigel (Biotechnology, Shanghai, China). Transfected MH7A cells (4 × 10^5^ cells/mL) in RPMI-1640 medium were seeded into the upper chamber of a transwell insert according to the manufacturer's instructions. RPMI-1640 medium (500 *μ*L) containing 10% FBS was added to the lower chamber. After 48 h, the cells on the upper side of the membrane were removed using a cotton swab, followed by multiple washes with phosphate-buffered solution. The cells on the lower surface of the membrane were fixed with 4% paraformaldehyde for 20 min and stained with 0.2% crystal violet (Biotechnology, Shanghai, China) for 15 min. An inverted microscope (Nikon, Tokyo, Japan) was used to capture the images, and the cells were counted.

### 2.8. Enzyme-Linked Immunosorbent Assay (ELISA)

After transfection for 48 h, MH7A cells were collected and cell culture medium samples were obtained. The corresponding ELISA kits (Abcam, Cambridge, UK) were used to test the concentrations of the cytokines (MMP1, ab100603; MMP3, ab100605; IL-6, ab46042; and IL-8, ab46032), following the manufacturer's instructions. The absorbance was monitored at 450 nm using a microplate reader.

### 2.9. Western Blotting

The cells were collected and lysed in radioimmunoprecipitation assay (RIPA) buffer (Beyotime) containing protease and phosphatase inhibitors on ice for 30 min. Protein concentration was determined using a BCA Protein Assay kit (Takara). Next, 50 *μ*g of proteins was separated on 10% sodium lauryl sulphate-polyacrylamide gels (SDS-PAGE) and transferred onto polyvinylidene difluoride (PVDF) membranes (Thermo Fisher Scientific). The membranes were blocked with 5% nonfat dry milk, and primary antibodies against ST3GAL3, cyclinD, cyclinE, PCNA, and GAPDH (1 : 1000, Abcam) were added overnight at 4°C. After three washes with Tris-HCl-buffered saline with 0.1% (*v*/*v*) Tween 20 (TBST), the membranes were incubated with anti-mouse horseradish peroxidase- (HRP-) linked secondary antibodies (Beyotime) for 1 h at room temperature. The intensity of protein expression was measured using an enhanced chemiluminescence reagent (Thermo Fisher Scientific).

### 2.10. Statistical Analysis

GraphPad Prism 6 (GraphPad Software, San Diego, USA) was used to analyse the data. The comparison between the two groups was performed using a paired *t*-test. The comparison between three or more was performed using one-way analysis of variance (ANOVA) with Tukey post hoc analysis. The results were expressed as mean ± standard deviation (SD). Each experiment was repeated three times in triplicate. *p* < 0.05 was considered to be significant.

## 3. Results

### 3.1. ST3GAL3 Was Upregulated in RA Tissues and Cell Lines

**Figure 1:**
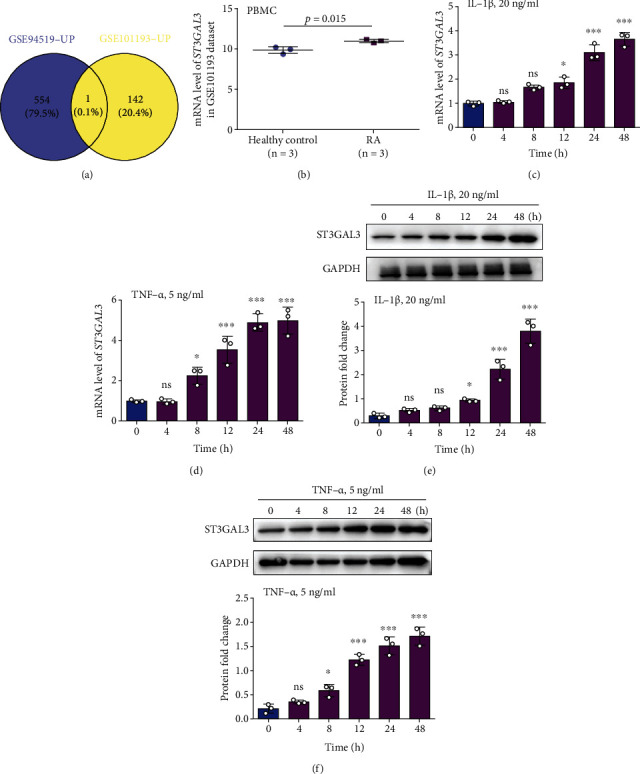
ST3GAL3 was upregulated in RA tissues and cell lines. (a) *ST3GAL3* was an overlapping gene screened from GSE101193 and GSE94519 datasets. (b) *ST3GAL3* was significantly highly expressed in the PBMCs of RA patients in contrast to normal controls in GSE101193 dataset. (c, d) The qRT-PCR and (e, f) western blotting results showed that the expression of ST3GAL3 was increased with prolonged IL-1*β* and TNF-*α* treatments in MH7A cells. *n* = 3. ns: no significance; ^∗^*p* < 0.05 and ^∗∗∗^*p* < 0.001 compared with the expression of ST3GAL3 at 0 h.

We compared the RA serum tissue expression datasets (GEO accession GSE94519) with the RA PBMC expression dataset (GEO accession GSE101193) from GEO. As shown in Figure 1(a), among the upregulated genes, *ST3GAL3* was an overlapping gene between the two microarrays. To further elucidate the molecular mechanism of *ST3GAL3* in RA, the GEO database was used to analyse the expression of *ST3GAL3*. The results showed that *ST3GAL3* expression was significantly elevated in RA tissues compared to normal tissues (*p* < 0.05, Figure 1(b)). We also examined the expression of ST3GAL3 in TNF-*α*- or IL-1*β*-induced MH7A cells. The results of qRT-PCR and western blotting showed that ST3GAL3 expression in inflammatory synovial cells significantly increased with prolongation of IL-1*β* and TNF-*α* treatments (*p* < 0.05, Figures 1(c)–1(f)). These results indicate that high ST3GAL3 expression may be involved in the development of RA.

### 3.2. ST3GAL3 Promoted the Proliferation and Migration of MH7A Cells

**Figure 2:**
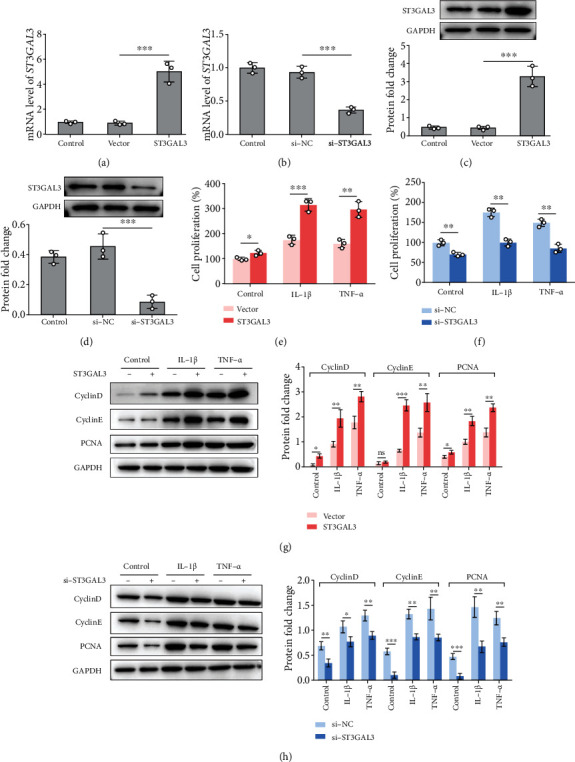
ST3GAL3 promoted the proliferation of MH7A cells. (a, b) After MH7A cells were transfected with ST3GAL3 overexpression plasmid or ST3GAL3 siRNA, qRT-PCR was performed to show the gene expression of *ST3GAL3*. (c, d) Western blotting analysis was performed to show the protein expression of ST3GAL3. (e, f) MTT assay showed that ST3GAL3 overexpression promoted cell proliferation, while si-ST3GAL3 inhibited cell proliferation. (g, h) Western blotting analysis showed that the overexpression of ST3GAL3 promoted the protein expression of cyclinD, cyclinE, and PCNA, while si-ST3GAL3 inhibited the expression of those proteins. *n* = 3. ^∗^*p* < 0.05, ^∗∗^*p* < 0.01, and ^∗∗∗^*p* < 0.001 compared with the indicated group.

**Figure 3:**
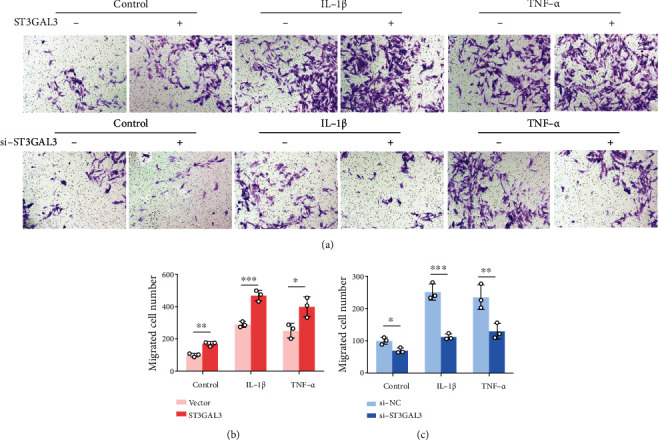
ST3GAL3 promoted the migration of MH7A cells. (a) Representative images of cell migration from transwell assay. (b) Transwell results showed that ST3GAL3 overexpression promoted cell migration, (c) while si-ST3GAL3 inhibited cell migration. *n* = 3. ^∗^*p* < 0.05, ^∗∗^*p* < 0.01, and ^∗∗∗^*p* < 0.001 compared with the indicated group.

To further verify the specific functions of ST3GAL3 in regulating the biological properties of MH7A cells, IL-1*β*-/TNF-*α*-induced MH7A cells were transfected with ST3GAL3 overexpression plasmids or ST3GAL3 siRNA. The gene and protein expressions of ST3GAL3 were both significantly upregulated by ST3GAL3 overexpression, and both were significantly downregulated by si-ST3GAL3 in MH7A cells compared to the vector control or si-NC group (*p* < 0.05, Figures 2(a)–2(d)). The proliferation of MH7A cells was detected using the MTT assay. The results showed that IL-1*β* and TNF-*α* treatments of MH7A cells resulted in a remarkable increase in cell proliferation. ST3GAL3 overexpression further increased the proliferation of IL-1*β*- and TNF-*α*-treated MH7A cells compared to the empty vector group (*p* < 0.05, Figure 2(e)), whereas ST3GAL3 siRNA inhibited the proliferation of IL-1*β*- and TNF-*α*-treated MH7A cells compared to the scrambled control group (*p* < 0.05, Figure 2(f)). Furthermore, the protein expression of proliferation-associated factors in transfected MH7A cells was detected using western blotting. The result showed that overexpression of ST3GAL3 promoted the protein expression of cyclinD, cyclinE, and PCNA (*p* < 0.05, Figure 2(g)), whereas ST3GAL3 siRNA had opposite effects on these proteins (*p* < 0.05, Figure 2(h)). We also detected the migration of MH7A cells using a transwell assay. IL-1*β* and TNF-*α* treatments of MH7A cells led to a significant increase in the number of migrated cells. The number of migrated cells further increased in the ST3GAL3 overexpression group (*p* < 0.05, Figures 3(a) and 3(b)) and decreased in the ST3GAL3 siRNA group (*p* < 0.05, Figures 3(a) and 3(c)) compared to the corresponding control groups. Therefore, ST3GAL3 promotes the proliferation and migration of MH7A cells.

### 3.3. ST3GAL3 Promoted the Production of MMPs and Inflammatory Factors

**Figure 4:**
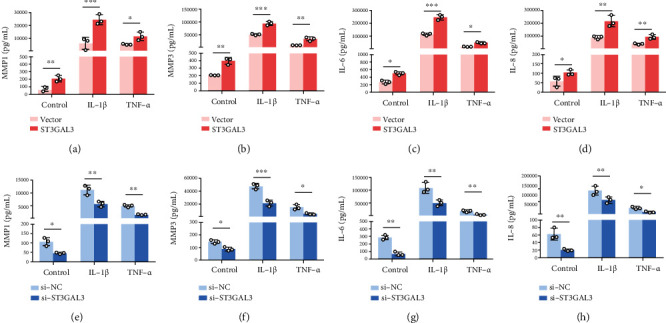
ST3GAL3 promoted the production of MMPs and inflammatory factors. (a–d) ELISA results showed that ST3GAL3 overexpression promoted MMP1, MMP3, IL-6, and IL-8 levels. (e–h) ELISA results showed that si-ST3GAL3 inhibited MMP1, MMP3, IL-6, and IL-8 levels. *n* = 3. ^∗^*p* < 0.05, ^∗∗^*p* < 0.01, and ^∗∗∗^*p* < 0.001 compared with the indicated group.

The anomalous secretion of inflammatory cytokines and degradative enzymes is vital to the progression of RA. The concentrations of inflammatory factors and MMPs in the supernatant of MH7A cells were measured using ELISA. IL-1*β* and TNF-*α* treatments are able to increase to a great extent the levels of MMPs and cytokines. ST3GAL3 overexpression further increased the levels of MMP1, MMP3, IL-6, and IL-8 (*p* < 0.05, Figures 4(a)–4(d)), whereas ST3GAL3 siRNA remarkably decreased the levels of IL-1*β*- and TNF-*α*-treated MH7A cells (*p* < 0.05, Figures 4(e)–4(h)). Thus, ST3GAL3 promotes the production of MMPs and inflammatory factors. These results suggest that ST3GAL3 promotes the production of MMPs and inflammatory factors in MH7A cells.

### 3.4. ST3GAL3 Regulated the TLR9/MyD88 Signalling Pathway

**Figure 5:**
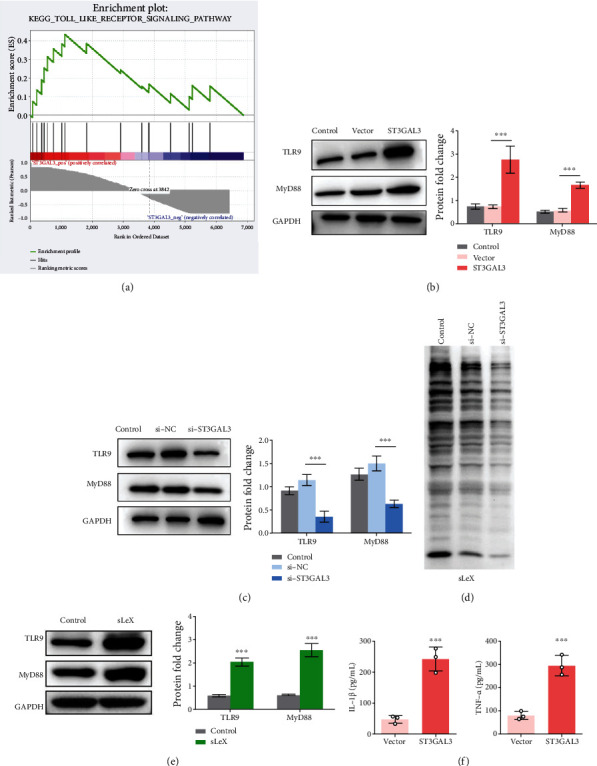
ST3GAL3 regulated the TLR9/MyD88 signalling pathway. (a) GSEA software was used to analyse the enrichment signalling pathways associated with *ST3GAL3* in RA. TLR signalling pathway was finally screened out. (b) Western blotting results showed that ST3GAL3 overexpression promoted the expression of TLR9 and MyD88. (c) Western blotting results showed that si-ST3GAL3 inhibited the expression of TLR9 and MyD88. (d) Western blotting result showed that si-ST3GAL3 inhibited sLeX levels in MH7A cells. (e) Western blotting result showed the activated TLR9/MyD88 signalling pathway by adding with sLeX in MH7A cells. (f) ELISA results showed that ST3GAL3 overexpression increased IL-1*β* and TNF-*α* levels. *n* = 3. ^∗∗∗^*p* < 0.001 compared with the indicated group.

To explore the latent mechanisms of *ST3GAL3* in RA progression, GSEA was conducted, and the TLR signalling pathway was finally screened (Figure 5(a)). Considering the importance of the TLR signalling pathway in regulating immune and inflammatory processes in RA, this pathway was selected for further research [24–26]. The protein levels of TLR pathway enrichment factors (TLR9 and MyD88) were detected using western blotting. The results presented in Figures 5(b) and 5(c) showed that, compared to the control, ST3GAL3 overexpression was significantly promoted, while si-ST3GAL3 notably inhibited the protein expression of TLR9 and MyD88 (*p* < 0.05). These results suggest that ST3GAL3 regulates the TLR9/MyD88 signalling pathway in MH7A cells.

Considering ST3GAL3 is a pivotal enzyme in the biosynthesis of sLeX oligosaccharides, the levels of sLeX in ST3GAL3-silenced cells were investigated.  Figure 5(d) shows that, compared to si-NC, silencing of ST3GAL3 remarkably reduced the sLeX levels. Addition of sLeX in the culture medium of MH7A cells significantly activated TLR9/MyD88 signalling pathway, as observed by the upregulation of TLR9 and MyD88 (*p* < 0.05, Figure 5(e)). These data suggested that ST3GAL3 increased sLeX levels, which further activated TLR9/MyD88 signalling pathway in MH7A cells. In addition, overexpression of ST3GAL3 in MH7A cells could also alter IL-1*β* and TNF-*α* levels (*p* < 0.05, Figure 5(f)).

### 3.5. ST3GAL3 Promoted the Inflammatory Response of MH7A Cells by Activating the TLR9/MyD88 Pathway

**Figure 6:**
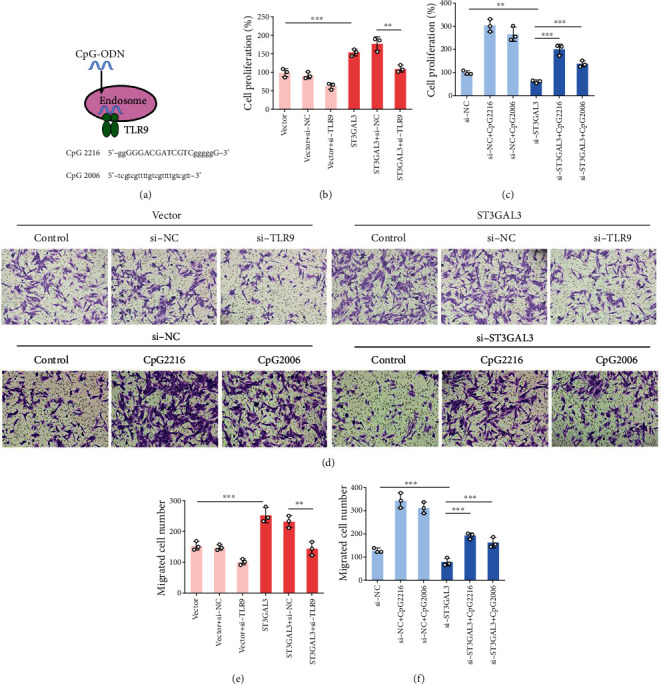
ST3GAL3 promoted the proliferation and migration of MH7A cells by activating the TLR9/MyD88 pathway. (a) The sequences of CpG-ODNs (CpG 2216 and CpG 2006). (b) MTT assay results showed that transfection of MH7A cells with si-TLR9 reversed the effects of ST3GAL3 on cell proliferation. (c) MTT assay results showed that adding with CpG-ODNs reversed the effects of si-ST3GAL3 on MH7A cell proliferation. (d) Representative images of cell migration from transwell assay. (e) Transwell assay results showed that transfection of MH7A cells with si-TLR9 reversed the effects of ST3GAL3 on cell migration. (f) Transwell assay results showed that adding with CpG-ODNs reversed the effects of si-ST3GAL3 on MH7A cell migration. *n* = 3. ^∗∗^*p* < 0.01 and ^∗∗∗^*p* < 0.001 compared with the indicated group.

To confirm whether ST3GAL3 affected the biological behaviour of IL-1*β*-induced MH7A cells through the TLR9/MyD88 pathway, we performed rescue experiments. TLR9 agonists (CpG-ODNs: CpG 2216 or CpG 2006; Figure 6(a)) were used in MH7A cells transfected with si-ST3GAL3 or si-NC. In addition, MH7A cells were transfected with ST3GAL3 overexpression plasmid, si-TLR9, or both. The MTT assay results showed that the addition of si-TLR9 significantly reversed the promoting effects of ST3GAL3 overexpression on MH7A cell proliferation (*p* < 0.05, Figure 6(b)). CpG-ODNs significantly reversed the inhibitory effects of si-ST3GAL3 on MH7A cell proliferation (*p* < 0.05, Figure 6(c)). Transwell assay results showed that the addition of si-TLR9 reversed the promoting effects of ST3GAL3 overexpression on MH7A cell migration (*p* < 0.05, Figures 6(d) and 6(e)). Consistently, CpG-ODNs reversed the inhibitory effects of si-ST3GAL3 on MH7A cell migration (*p* < 0.05, Figures 6(d) and 6(f)).

**Figure 7:**
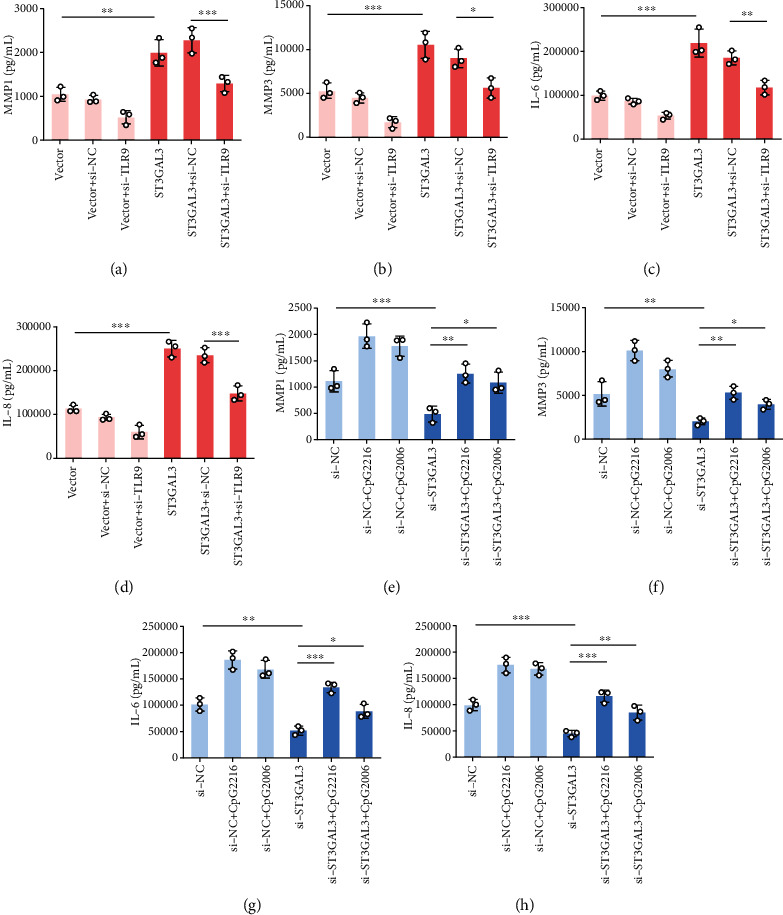
ST3GAL3 promoted the inflammatory response of MH7A cells by activating the TLR9/MyD88 pathway. (a–d) ELISA results showed that transfection of MH7A cells with si-TLR9 reversed the effects of ST3GAL3 on MMP1, MMP3, IL-6, and IL-8 levels. (e–h) ELISA results showed that adding with CpG-ODNs reversed the effects of si-ST3GAL3 on MMP1, MMP3, IL-6, and IL-8 levels. *n* = 3. ^∗^*p* < 0.05, ^∗^*p* < 0.01, and ^∗∗∗^*p* < 0.001 compared with the indicated group.

Finally, ELISA results showed that the increase in MMP1, MMP3, IL-6, and IL-8 levels in ST3GAL3-overexpressing cells was reversed by transfection with si-TLR9 (*p* < 0.05, Figures 7(a)–7(d)). The decrease in MMP1, MMP3, IL-6, and IL-8 levels in ST3GAL3-silencing cells was reversed by adding CpG-ODNs (*p* < 0.05, Figures 7(e)–7(h)). Therefore, ST3GAL3 promotes the inflammatory response of MH7A cells by activating the TLR9/MyD88 pathway.

## 4. Discussion

As a systemic autoimmune inflammatory disease, RA principally affects synovial joints and is considered a high-risk factor for cardiovascular disease [27]. Alteration of gene expression has been identified as an important factor in the pathogenesis of RA [28]. Although a great deal of work has been done to confirm or evaluate specific genes and pathways involved in the progression of RA, there is still a lack of research on effective genes and their mechanisms in RA. Abnormal expression of ST3GAL3 develops in many diseases, especially human cancers [18]. Furthermore, the synergistic effect of ST3GAL3 and IL-1*β* has significant toxic and adverse effects on pancreatic tumour [19]. Because the expression level of ST3GAL3 in RA has not been studied previously, we first investigated the expression of ST3GAL3 in RA. Bioinformatics analysis showed that *ST3GAL3* was highly expressed in the PMBC of patients with RA.

Considering that FLS can be activated by TNF-*α* and IL-1*β* and exhibit biological behaviours similar to tumour cells [11, 12], we used IL-1*β* and TNF-*α* to treat human RA-derived FLS MH7A cells to construct RA cell models. We found that the expression of ST3GAL3 in MH7A cells increased with prolonged IL-1*β* and TNF-*α* treatments, which indicated successful cell modelling. To investigate the role of ST3GAL3 in the progression of RA, MH7A cells were transfected with the ST3GAL3 overexpression plasmid or ST3GAL3 siRNA. Joint swelling and synovial hyperplasia are prominent characteristics of RA, which are induced by the inflammatory proliferation of synovial cells, especially FLS [29]. The results of the MTT assay and western blotting showed that silencing ST3GAL3 markedly inhibited the proliferation of RA-FLS, suggesting that ST3GAL3 might have significant regulatory effects on the growth of RA-FLS. Bone destruction and cartilage erosion are two major histopathological features of RA [30]. The precocious inflammatory microenvironment can fuel the migration and invasion of FLS to the internal structure of the joint, which eventually results in injury to the subchondral bone and cartilage [31]. This indicates that the migration and invasion of RA-FLS are likely to be the main changes in the pathogenesis of RA. In this study, transwell analysis showed that the overexpression of ST3GAL3 promoted the migration of cells with TNF-*α*-/IL-1*β*-induced inflammation, whereas ST3GAL3 silencing had the opposite effect.

Activated FLS can act as a pivotal regulator of the synovial inflammatory response in RA. RA-FLS generally secrete a variety of proinflammatory cytokines and chemokines such as IL-6, IL-8, and MMPs, which recruit and activate more immune cells into the inflammatory microenvironment, leading to destruction of cartilage and joints [32]. Therefore, if the production of those inflammatory factors is suppressed, signs and symptoms of RA may be expressively ameliorated [9, 10]. In our study, overexpression of ST3GAL3 promoted the secretion of MMPs (MMP1 and MMP3) and inflammatory factors (IL-6 and IL-8) in the *in vitro* model of RA. By contrast, ST3GAL3 silencing reversed these effects. These results indicated that ST3GAL3 actively regulates the proliferation, migration, and inflammation of FLS *in vitro*. Overexpression of ST3GAL3 in RA-FLS may lead to an increase in rheumatoid synovial hyperplasia and aggression, eventually resulting in joint destruction. In addition, increasing ST3GAL3 in MH7A cells also alters the levels of TNF-*α* and IL-1*β*. The feedback regulation further increased the inflammatory progression of RA.

TLRs are innate immune receptors. Recent studies have indicated a key role of TLRs in the formation of an adaptive immune response, which provides potential therapeutic value for disease treatment [15, 33]. Furthermore, in the human RA-FLS cell line MH7A, TLRs can stimulate the activation of cytokines, including IL-6 and TNF-*α*, which are pivotal mediators of inflammation and could also induce chemokines of acute inflammation [15]. Most TLR signals function via the MyD88-dependent pathway. Huang and Yang confirmed the role of the TLR9/MyD88 pathway in the regulation of adaptive immune responses in autoimmune diseases [34]. However, the effects of the TLR9/MyD88 pathway have never been studied on RA progression. In this study, TLR9/MyD88 was found to be a downstream activator of ST3GAL3. To verify the mechanism of the interaction between ST3GAL3 and the TLR9/MyD88 pathway in the progression of RA, several validation experiments were conducted. Transfection of cells with TLR9 siRNA reversed the promoting effects of ST3GAL3 on MH7A cell proliferation, migration, and inflammation. Addition of the TLR9 agonist CpG-ODNs reversed the effects of ST3GAL3 silencing on MH7A cell proliferation, migration, and inflammation. These results suggested that ST3GAL3 contributes to the FLS inflammatory response in RA by activating the TLR9/MyD88 pathway. In addition, we found that silencing of ST3GAL3 in MH7A cells reduced the levels of sLeX, which was able to active TLR9/MyD88 pathway.

There are several limitations in the present study. (1) Despite this study revealed the regulation between ST3GAL3 and sLeX in MH7A cells, further studies are required to confirm its regulation *in vivo*. (2) RA is characterised by the activated lymphocytes [35] and the differentially expressed biomarkers in the serum and PBMC [36, 37]. Here, we observed the increased expression of ST3GAL3 in the serum and PBMC of RA patients from GEO datasets. But the effects of ST3GAL3 on PBMC or lymphocytes were not revealed. (3) It is unclear whether ST3GAL3 exerts its effects on normal FLS. A lot of studies are required to determine the complexity of ST3GAL3 in promoting the progression of RA.

In conclusion, ST3GAL3 was highly expressed in RA-FLS. ST3GAL3 actively regulated the proliferation, migration, and inflammation of the RA-FLS cell line MH7A by activating the TLR9/MyD88 pathway, which was involved in RA pathogenesis. Thus, ST3GAL3 may serve as a novel therapeutic target for RA.

## Data Availability

The datasets used and analysed during the current study are available from the corresponding author.
